# Psychotherapeia, or the Remedial Influence of Mind

**Published:** 1853-04-01

**Authors:** W. C. Dendy

**Affiliations:** AUTHOR OF THE "PHILOSOPHY OF MYSTERY," &c.


### PSYCHOTHERAPEIA, OR THE REMEDIAL INFLUENCE OF MIND.

BY W. C. DENDY, ESQ., AUTHOR OF THE "PHILOSOPHY OF MYSTERY," &c.*

WHEN Plato wrote these words—"nec totum corpus (curabis) sine animâ," he recorded a truth which few probably will deny, but the principle of which, in the practice of medicine, has been constantly blinked or set aside. This error has been committed, not only from deficient appreciation of the influence of mind, and especially that one of its faculties we term *volition*; but also from a notion that the psychologist speaks and writes of intellect as an abstraction, and not as that intimate union of mind and matter which has laid the basis of modern psychology, and especially of the theory of insanity. What the blood is to a secreting gland the spirit is to the brain—the gland forms its especial product from blood; the brain acting with spirit, so to write, produces mind. Now, whatever the nature of this union may be, we know there is a constant reciprocity or mutual influence between the two elements: and to show how mind acts on tissue let us take the course of a simple thought, the subject of which is sufficiently potent to cause *sensible effects*; we may call it emotion. The sensations it often induces are those which if in greater degree or more permanent, would be the very symptoms or indications of disorder. What is a chill (as of fear), but a rigor, like that of ague, and its cause is cardiac congestion. What a throb, but that exalted innervation, which if protracted would probably induce cardiac hypertrophy. What the flush, but that hyperæmal condition, which if not quickly subsiding might terminate in inflammation.

The true psychologist, therefore, discards metaphysics entirely from his vocabulary. With him, mind and brain indeed are almost convertible terms; their influence on the heart being almost instantaneous; a fact which has indeed caused that organ to be conventionally, though absurdly, referred to as the seat of the sentiments. It is true that the innervation of the heart is chiefly ganglionic, but its association with the brain, the power of will over even incident or reflex innervation, is proved by its obedience, as in the cases of Coma, of Fontana and Colonel Townsend: and the heart pays back this compliment in kind: Dr. Wardrop enumerating twenty disorders which result at once from this mutual influence of brain and heart. We know that this influence also is both special and common; if thought be concentrated on one organ, it may there at once induce an especial disorder, or by affecting the heart itself primarily, it may soon derange the condition of the whole vascular system. Intense emotion, even constant thought, will often disorganize the cerebral tissue, and disease of the brain may gradually derange or instantly annihilate the manifestations of the mind.

The pathological influence of mind is as deeply interesting as it is evident on the structures of the body. The effect is often as it were electric, altering at once not only the feelings but the secretory apparatus of an organ; the colour of the hair has been changed from black to grey, even in a few hours, as in the case of the young Sardinian fowler, and of Marie Antoinette, whose beautiful locks, it is stated, became almost white during her return from Varennes to Paris. The same is stated of Lebeny, the man who stabbed the Emperor of Austria a short time ago. At other times the constant anticipation or foreshadowing of a coming evil will often reduce the system so much as to incapacitate it for bearing that evil with impunity. Cases are recorded, by Mr. Travers and others, of patients having either dreamed of the fatal result of an operation, or brooded over its perils, and *thence* dying soon after

* Read, March 12, before the Medical Society of London.

its performance, every step of which was seemingly propitious. *Shock* will often at once strike down as it were the very life of a being, inducing syncope, trance, or epilepsy: or by a more severe mental blow on the brain, the organic power may be permanently paralyzed, and death be the result. A few years ago, just previous to the death of Sir Astley Cooper, he was called in to reconcile the difference of opinion between another surgeon and myself, regarding the propriety of operating on the scirrhous breast of a lady who came from the country, not to *consult* me regarding her malady, but to request me to operate on her at once. Her expressions were most cheerful, and she was evidently buoyed up by a confident hope of being speedily relieved by the operation. On Sir Astley's announcing somewhat abruptly his disapproval of the operation, the lady almost started from her seat, and soon after fainted. From the moment of return of consciousness despondency took possession of her mind, and gradually declining she sank in three weeks from the delivery of the verdict.

The effect of fear we know will be frequently to induce diuresis and diarrhœa; anger and jealousy will soon clog the bile ducts, and originate jaundice and melancholy. Murat was directly in a state of jaundice if he heard bad news from Naples, while in Russia. One of my mercantile friends almost invariably dislodged from his stomach the whole of a hearty breakfast, if on his adjournment to his counting-house he opened a letter containing accounts of any mishap to his freights or his ventures. The proper secretions, as that of milk, are constantly checked by grief: alarm and dread will suppress the salivary flow; a truth of which the Indian magician often takes an ingenious advantage in the discovery of a criminal. Mere anxiety also, by reducing the vital energy, will render the body at once prone to malarious infection, or parasitic development. Terror, even induced by illusion, may in a few moments prove fatal, as in the case of the criminal who died under the erroneous notion that he was being bled to death.

The sexual stimulus is constantly influenced by those feelings and emotions which interfere with or neutralize instinctive passion. An excess of esteem, as well as a doubt of virility, may destroy for a time the sexual energy; and I am aware of more than one instance in which the act of coition can only or more efficiently be performed in the morning, when consciousness, being lost in slumber, has left the spinal or animal influence unrestrained.

In the female, also, intensity of sensibility or sensation, or the hyperæsthesia of erotic passion, will in a moment induce syncope and impotence. Some years ago I was several times called in the night to a young married lady, who although having gone to bed well, was suddenly attacked by acute hysteria, the prominent symptom of which was complete aphonia. The period was midnight, and as I learned in confidence, the paroxysm came on at the onset of the connubial embrace of her husband. Valerian with anodynes, and temporary sexual abstinence, though not separation, were the remedies.

The chronic yet woeful effects of the overwrought mind are multiform. How many are the melancholy instances of suicide in the subjects of overstrained genius. Remember Ariosto, Collins, Cowper, White, Byron, Coleridge, Paganini, Malibran; the spirit of each might exclaim with Manfred—

> "Look on me—there is an order
> Of mortals on the earth who do become
> Old in their youth, and die in middle age,
> Without the violence of warlike death;
> Some perishing of study—
> And some insanity."

In this penalty of genius we see, however, the balance of happiness beautifully adjusted; the exaltation of mental as of corporeal pleasure being followed by despondency and peril. Mind is in these instances a hard and cruel master, but by discipline and culture it may often be made a valuable servant.

The psychological and prophylactic, and, may we add, therapeutic influence of the mens sana are as clear as the pathological effect of mind. It would be easy to fill many pages with illustrations of this truth : it is of course these influences which constitute the remedial powers of mind even when disorder is established. It is often deeply interesting to mark the salutary changes which result from the influence of a devout and philosophic spirit, and also of the lighter and more joyous states of the mind when brought to play even on structural disease. As we know that mental states induce disorder, we may also perceive, that prevention and cure may be effected simply by inducing a contrary condition of mind. A sthenic disorder excited by excess of emotion will often subside on the supervention of an asthenic state of the mental organ. Even the secreting tissue may be obedient to this principle, the whitening of the hair may subside on the removal of its cause of fear or grief; the reduction of hernia has been easily effected when the body is under the depressing influence of alarm.

The principle of John Hunter may thus be applied even to psychology—one thought displacing another, and it were not difficult to construct on this basis an allopathic table of psychological antagonisms—opposing, for instance, the effects of anxiety, or pride, fear, melancholy, envy, hatred, remorse, by devotion, cheerfulness, self-control, piety: *contraria contrariis curantur.*

In following up this argument we cannot, I think, deny a certain influence of other minds on our own, although the real truths are so unblushingly warped and exaggerated to favour the views of the empirical impostor. What was the principle of tractors—of potions—of electro-biology—of the shampooing of Valentine Greatrex, but the effect of mental impression ; a change nervous and vascular is induced, and its consequence must be some change of action, it *may* be morbid. An acknowledgment of this truth would soon take the remedy of mental influence from the hands of the impostor, and gain for us a valuable aid in our ministration.

I was some time ago attending a young lady with typhoid fever, to the friends of whom one of the most notorious mesmerizers had been strongly recommended ; indeed he was brought to the house during one of my visits. I was not at all reluctant to argue the question, and my arguments prevailed of course with the enlightened members of the family ; but for fifteen minutes while I was explaining, and indeed convincing as I believed, the professor was playing a deep game with me. In profound silence and abstraction he fixed his hawk's eye on mine, and I confess and declare that the sensations of extreme heat and something like vertigo caused me no slight fear, lest I should in the end be practically floored by my antagonist. It was evidently his scheme to put himself, as he would call it, *en rapport* with me.

When the mind is pleasurably excited, the emotion of joy, the circulation and innervation are of course more healthy. Even the organic functions dependent on spinal and ganglionic influence may be instantly excited. I had a patient in whom the peristaltic action was directly induced by a brief glance at the *Times* newspaper ; and I know a gentleman in whom the same effect instantly results from the study of a map ; it is very rare indeed that this expedient fails. We know, too, how instantaneously a thought will stimulate the salivary, the spermatic, and other glands.

Now as one of the immediate effects of grief or fear is, as we know, to reduce action and secretion, they might thus possibly be converted into a remedial agent in the suppression of hæmorrhage, and also in those cases of acute neuralgia which depend on plethora or increased determination, as inflammatory toothache, &c. It is by the production of analogous sensations that remedial effects are induced by the hand of the hanging criminal, the drinking of warm blood, the toad amulet, &c. Probably the sense of shame may thus be auxiliary in the removal of internal hyperæmia by the rush of blood to the surface of the body—counteraction or derivation.

When, however, this emotion of fear is heightened into terror, very opposite and most eccentric consequences may be produced. The previously speechless son of Crœsus is recorded by Herodotus to have exclaimed, "Kill not Crœsus," on the uplifting of the assassin's arm; and Battus, according to Pausanias, recovered his lost speech at the sight of a lion at his side.

The contrasts of fear are hope, faith, confidence. As hope casts a *couleur de rose* over the heart and mind, faith and confidence will often effect more for disorder than a bevy of physicians with the whole materia medica at their command. Yet how is this influence disregarded in practice. For hope is not only *felt* in the heart, but it is synchronously the immediate cause of a vigorous circulation. It is recorded, on the contrary, how deeply the circulation and energy of the soldier are affected, so soon as the army turns on its inglorious retreat. The pulse is irritable and languid, the respiration slower and irregular, and the asthenia of disappointment at once sets in. In the hospital of a defeated army the healing process is far more slow and imperfect than in that of the conquerors. And why is this? The thought in the brain at once oxygenizes the blood in the first case, and carbonizes it in the other; the extreme of these states being liable to rise or lapse into conditions of inflammation or melancholy: these contrasted phenomena have been indeed noticed in the same subject. The drivelling idiot has, under acute cerebral fever, as the excited circulation has lighted up the brain, become half rational for a time; that which would by excess make another mad brings out into relief his asthenic or apathetic intellect, which again dwindles as the action subsides.

In obstetrics this is daily proved. We know, too, that as the sudden entrance of a strange accoucheur will instantly annihilate the parturient effort, so the arrival of the favourite doctor will directly set all the functions going again.

In Lord Anson's voyage, despondency and hope were proved to be the exciting cause and remedy in the most malignant attacks of scorbutus. And in that most severe epidemic scurvy, at the siege of Breda, the pious fraud of the Prince of Orange in vaunting the miraculous powers of an elixir really of the most simple composition, very speedily, by the imparting of hope and confidence, established healthy action, and cured the patients who had been for months completely disabled.

Joy, the contrast of grief, is of course a feeling of still deeper intensity, and the wisdom of Solomon was aware of its salutary influence, when he wrote the proverb—"A merry heart is the life of the flesh." Yet excess of joy may madden or kill; insanity has often been induced by sudden accession of property, and the widow fell dead on the unexpected return of her son. I was some time ago one of a long list of doctors who had endeavoured in vain to restore the power of speech to a young lady, who had for many months been afflicted with hysterical aphonia. During this course she was promised one of the jewels in the Exhibition if she would pronounce its name; with extreme effort she gained her prize, but the *strain* directly aggravated the malady for some time afterwards. The nearest approximation to remedy or cure was effected at last by the cold water douche, as a forlorn hope, the essence of which was, I believe, as much shock as the refrigerating influence of the cold fluid.

True love is the highest, deepest, and holiest source of joy, as it is the most unselfish.

Blighted love and jealousy constitute the most fertile sources of indisposition,—"the worm i' the bud" which foils our study and efforts in the cases of chorea, hysteria, amenorrhœa, and melancholy, and even the development of intellect.

Mutual affection, or happy love, is at once its antidote. Even in a few hours, we have probably all known the protean symptoms of organic asthenia, as well as of psychical depression, disappear as if by the spell of an enchanter; and all this from the mere assurance in the mind of a woman that she is beloved. The remedial influence of mind is in nothing more immediate or striking than in this. A reprieve has often been granted even at the eleventh hour. The physician is constantly consulted in the cases of young women, in which he sees at once the remedy, but of which he cannot propose the adoption. The mental counteraction of the more violent *passions* may often effect a very sudden cure. Van Swieten records the sudden relief of acute gout by extreme fright induced by a ghost; and Haller, from a violent paroxysm of anger; and Valerius Maximus, from the same cause and its consequence—increased innervation, even to the restoration of a paralytic limb. We are all aware of the instant alleviation of an excruciating toothache by the mere touch of a dentist's rapper.

One of the most prevalent errors of the human mind consists in the conception of wrong notions of one's-self. It has passed into a proverb, e cœlo descendit, γνωθι σεαυτον: but how rarely, if ever, does this divine emanation find entrance into the heart or mind. The world will scarcely allow it. But the principle obtains as well in physics as in morals. Like evil thoughts, the illusive belief that disease exists in a part, will sometimes, by concentration of nervous and muscular energy, so influence the body as to become a very fertile source of indisposition; and it may, indeed, in time even induce the very organic disease which it had merely imagined.

It is in hysteria especially that this *auto*-mania, or morbid thinking of one's-self, chiefly occurs; although it is probable that few are altogether without it. To one lady especially would I allude, who came under my care for acute hysteria—the surface of whose body, the abdomen especially, was so intensely sensitive, that a feather dropped on it caused her to scream with agony — nay, even the approach of the finger would induce an extreme degree of this hyperæsthesia. No medicine was of avail, but her great relief was procured by psychical treatment alone. Although a sudden touch was then agonizing, delicate and gradual pressure was soon borne without suffering, and the mind being brought to think rightly of the nature of her malady, the lady was relieved at least of one severe affection.

Somewhat of this nature are the cases of hysterical spine and knee, as they are termed, especially by Brodie and Todd. These two neuroses are particularly distinguished from inflammatory affection, by the sleep of the patient being undisturbed, whereas in structural disease the patient is constantly awoke by pain during the night.

I believe that if, on this principle, the current of morbid innervation be intercepted or kept in abeyance long enough, by insensibility, or slumber, or even by protracted diversion, many of the neuroses might be thus alleviated or dispelled. The mind would forget the malady, and it would cease, according to the Berkleyan theorem, to exist.

I am now attending a lady in Camden villas, whose sensory condition is most eccentric — probably illusory. She believes that the most offensive odours issue from her mouth and nose, although her husband, of course a very competent judge, is perfectly convinced to the contrary. De Boismont refers to the case of Madame L., who was affected by this depraved æsthesia; she sniffed the most disgusting odours with perfect ecstasy. If my own patient could sleep for a week, and the mind lie perfectly fallow, she would, I think, be well. Closely allied to this fallow of the mind is the state of insanity, which is often a prophylaxis, and a remedy for those physical derangements which commenced in the previous condition of sanity. The lunatic's mind is almost a tabula rasa, and not thinking of danger, it is also so far capable of resisting the impression of malarious influence. I adduce these negative states of mental etiology, to point thus, by the way, to the obvious mode of treating these illusions.

On this point I may affirm that agreeable deception, nay, that which may be termed a pious fraud, may be conscientiously and most beneficially adopted in many cases of hysteria. The supposed morbid *effects* of an easterly wind have been really averted from the hypochondriac by nailing the vane to the westerly point. And patients who have imagined that they carried within them the most monstrous entozoa, have been cured by an emetic, something resembling the parasitic monster having been, previously to its effect, secretly placed in the basin.

The miracles of Hohenlöhe were precisely on this principle. The extraordinary case recorded by Dr. Baddely of Chelmsford ceases to be a mystery, when we illustrate and explain it by the power of implicit confidence, consecrated as it were by intense devotion.

About three years ago, I myself proved the powerful prophylaxis of mental concentration on another point, during a most awful and perilous night voyage from Boulogne. There were ten gentlemen in the saloon, of whom eight were prostrate; and I should certainly have joined the interesting group, had I not fixed my thoughts intensely on the pictorial and sculptured treasures of the Louvre and Versailles.

I visited, some years ago, a lady in the west of Sussex, in whom intense hyperæsthesia of the skin was the torment of her life during the latter stages of pregnancy. Even while I was watching her, I observed that, while her attention was interestingly diverted from herself, she left off scratching. It is clear, therefore, that one prominent principle in these cases is mental counteraction. Once arrest the attention on any other subject than self, and we shall often see its remedial influence on that malady, which, as we have all proved, will constantly resist the laboratory and the materia medica. But the illusion *may be* so severe, as to amount to confirmed madness on one point, and this automania may lead to fatality. One of my medical friends had for some time laboured under the illusion that syphilitic caries of the nasal and palatine bones was rapidly progressing, and would destroy him. It was not difficult to reason him, at times, out of this phantasy; but he would, sooner or later, relapse. In the lucid intervals, he visited and prescribed with judgment and discretion; yet immediately after a day spent in professional duties, the wrong notion of self came across him; and in a moment he half divided very scientifically the brachial artery, and bled to death—*γνωθι σεαυτον* would have saved him from the act of suicide.

This morbid introspection, productive as it is of disorder, it is our duty to divert or set aside, as much as it is to watch and warn the brain and mammon slaves of *their* infatuation. Yet we go on pilling and draughting; at best, merely removing consequences, forgetful of the giant effects of the sympathies, and on the failure of our therapeutic efforts, wonder that the brain, heart, and lungs, should still become disordered or disorganized.

Intense thought and calculation had induced in Mr. M., a man of robust health, a throbbing and intermission of the pulse. Keeping the mind in fallow, or diversion, twice restored the heart's integrity. Immense speculations, and the *crisis* or *panic* concentrated his thought on his ledgers, and he entirely forgot himself and his functions. Mitral disease, hypertrophy, and *universal* effusion was the end of this; and from his right pleural cavity I drew off at once five pints of fluid. And all this might have been averted, had thought been diffused or diverted; or he had been dissuaded from this mismanagement of self.

In deep study, this concentration of thought is a constant source of self-forgetfulness. The heart being an involuntary muscle, will still act as well as congestion will allow it. But congested lung, when instinct fails in its duty, must be relieved by voluntary effort. We must not forget to breathe. The consequent collapse of the air-cells will not only increase congestion, but especially favour the development of tubercle. It is often by the due expansion of cells that the granule or germ in the pulmonary parenchyma is subdued or kept down. So that volition, or direction of mind to the pulmonary apparatus, so as to ensure full and deep breathing, may be not only remedial, but prophylactic of consumption itself. Indeed, we may believe that mysterious dissolution may be sometimes referred to this stealthy cause. The cases of Bateman and Hunter might have formed fatal illustrations, had not the one been almost incessantly roused from slumber, and the other set himself to deep and voluntary inflation of his lungs. On this principle, sleep is sometimes perilous in disorders of the pulmonary system, as it withdraws volition. Probably this may have been the immediate causa mortis in old asthmatic persons, who having long endured a sort of chronic atelectasis, have been discovered dead in their beds.

I have studied to limit, as much as possible, this crude paper, avoiding any direct allusion to the pathology of sympathetic and reflex actions, confining my remarks chiefly to the points of prevention and remedy by psychical influence. But this, of course, only through the medium of matter; for the metaphysical treatment of disorder would be an absurd solecism. The basis of my remarks is of course the proposition that a mere thought instantly induces a physical change, probably even in the condition of the blood, and that by the directing or averting such thought to or from disordered structure or function, we may constantly avail ourselves of a valuable auxiliary in the practice of our intricate science.

